# Outcome of Central Vein Occlusion Recanalization in Hemodialysis Patients and Predictors for Success: A Retrospective Study

**DOI:** 10.5334/jbsr.1991

**Published:** 2020-05-06

**Authors:** Keerati Hongsakul, Pattarasuda Leelarujijaroen, Ussanee Boonsrirat

**Affiliations:** 1 Prince of Songkla University, TH

**Keywords:** central vein occlusion, endovascular technique, success, patency rate, predictor, fistula

## Abstract

**Background:**

Catheter-directed treatment is the standard approach for the management of chronic central venous occlusion.

**Purpose:**

The objective of this study is to report the outcome of conventional recanalization of chronic central vein occlusion in hemodialysis patients and to determine the predictors for success.

**Material and Methods:**

All hemodialysis patients who underwent endovascular recanalization of central vein occlusion from January 2012 to December 2016 were retrospectively evaluated. The procedure was percutaneous transluminal angioplasty. Stenting was performed in case of a significant recoil stenosis. Kaplan-Meier analysis was used to evaluate central vein patency. Univariate analysis and multivariate logistic regression were used to calculate the predictive factors.

**Results:**

Ninety-seven patients (mean age, 61.2 years; range, 25‒89 years old) with 97 central vein occlusions were enrolled. Technical success was achieved in 49 patients (50.5%). The primary patency rates of central veins at 6 and 12 months were achieved in 17 patients (34.4%) and 8 patients (15.8%), respectively. The assisted primary patency rates at 6 and 12 months were achieved in 38 patients (77.3%) and 30 patients (61%), respectively. Patient age ≥60 years and a tapered-type of lesion were significant predictive factors for successful recanalization.

**Conclusion:**

Endovascular treatment of the central vein occlusion using a conventional technique is moderately effective and safe. Angioplasty alone and stenting were not significantly different in terms of patency rate. The age of the patients and type of occlusion were significant predictors for successful recanalization.

## Introduction

Central vein stenosis (CVS) and central vein occlusion (CVO) are common and important problems in hemodialysis patients that cause venous hypertension and access flow dysfunction, resulting in access recirculation and inadequate dialysis. The symptoms of CVS and CVO may include edema of the ipsilateral extremity, facial edema, development of collateral vessels at the chest wall, upper extremity and neck, and pleural effusion [1].

Endovascular intervention with percutaneous transluminal angioplasty (PTA) is the first-line treatment of CVS and CVO [2], with reported technical success rates ranging from 47% to 90% [3,4,5,6]. Stenting can provide mechanical support for a lesion that is unresponsive to PTA. It is beneficial in kinked stenosis, elastic recoil after balloon angioplasty (BA), sealing flow limiting dissection, and maintaining patency of chronic CVO [2].

Recanalization of CVO is technically difficult. The standard technique using a diagnostic catheter and guidewire is the primary option followed by BA. Other advanced techniques that use the stiff backend of a guidewire or long needles can be considered when the standard technique fails [7]. The previous literature reported the success and patency rates of endovascular treatment in a combination of cases of CVS and CVO [3,4,5,6,8,9,10,11]. There are no reported data on endovascular outcome in hemodialysis patients with CVO only, and the predictive factors were not determined for recanalization success.

In an attempt to evaluate the outcome of endovascular intervention in CVO in hemodialysis patients, we present our experience on CVO recanalization and determine the predictive factors for successful recanalization.

## Materials and Methods

### Patients

This is a retrospective study of consecutive hemodialysis patients who underwent endovascular intervention for recanalization of chronic CVO from January 2012 to December 2016 in Songklanagarind Hospital, which is a university hospital in southern Thailand. Ninety-seven hemodialysis patients with 97 chronic CVO are enrolled. This study was approved by the Ethics Committee of the Faculty of Medicine, Prince of Songkla University (IRB No. 60-259-07-4).

### Procedure

Informed consent was obtained from the patients before the procedure. Almost all of the procedures were performed while the patient is under local anesthesia. However, the procedure was performed in a few patients while they were under general anesthesia as they could not co-operate with the procedure under local anesthesia. Initially, the dialysis circuit was evaluated by Doppler ultrasound. An antegrade puncture was performed in the venous drainage of the arm using an 18-G needle, for the angiogram of the central vein. In the case of CVO, an 8-French (Fr) vascular sheath was inserted via the basilic or cephalic vein of the ipsilateral arm over a 0.035-inch hydrophilic guidewire. A total dose of 3000 IU of heparin was given via the vascular sheath. Then, recanalization of the occluded segment of the central vein was performed using a supported 4 Fr angiographic catheter (Bern, Boston Scientific, MA, USA) and a stiff hydrophilic guidewire (Radifocus®, Terumo, Tokyo, Japan). If the guidewire could pass the occluded segment into the right atrium, the angioplasty was performed using a non-compliant balloon (Mustang, Boston Scientific, MA, USA) with a diameter of 5 mm at normal pressure (10 atm) for 1min pre-dilatation. Then, an ATLAS® high pressure balloon catheter (BARD, AZ, USA) with a diameter of 12–14 mm was used to dilate the occluded segment at 6–10 atm until full expansion of the balloon for 2 min. If the guidewire could not pass the occluded segment via the antegrade approach, the combined retrograde approach via the right common femoral vein was performed using a 45-cm-long guiding sheath (Destination®, Terumo, Tokyo, Japan) to act as a supporting vascular sheath, and then recanalization was performed using similar devices and technique as those used in antegrade recanalization. A final angiogram was performed to evaluate the residual stenosis. In case of recoil stenosis greater than or equal to 30%, a self-expandable bare metallic stent (SMART, Cordis, FL, USA) with a diameter of 12–14 mm and length of 40–60 mm was placed. Post-stent BA was performed to fully expand the stent if needed. A post-stent angiogram was also performed to evaluate stent patency and residual stenosis. After completing the procedure, the vascular sheath was removed, and manual compression was applied until hemostasis was achieved.

### Follow-up

All successful recanalized patients underwent regular hemodialysis procedures, and all of them attended follow-up at the hemodialysis center and vascular surgery clinic. Cases of suspected significant restenosis of the central vein were referred to the interventional radiology unit for repeat angiogram and endovascular treatment.

### Definition

According to the reporting standards for percutaneous interventions in dialysis access published by the Society of Interventional Radiology [12], technical success is defined as a successful procedure with less than 30% residual stenosis. Primary patency is defined as the time interval between a successful initial procedure and the first repeat intervention or significant restenosis. Assisted primary patency is defined as the cumulative interval between all repeat interventions performed to maintain patency until placement of a new access site, abandonment of the access site, ligation of the access site, or placement of a dialysis catheter.

### Statistical analysis

Analyses were performed using R software Version 3.4.3. Categorical values were reported as frequencies or percentages. Continuous values were reported as means ± standard deviation. Patency rates were calculated using Kaplan-Meier analysis. Wilcoxon rank-sum test or Fisher’s exact test was used to evaluate any significant differences between categorical values. Student’s t-test was used to evaluate any significant differences between continuous values. Potential predictors for success were evaluated by univariate analysis and multivariate logistic regression. A P-value of less than 0.05 was considered a statistically significant difference.

## Results

**Figure 1:**
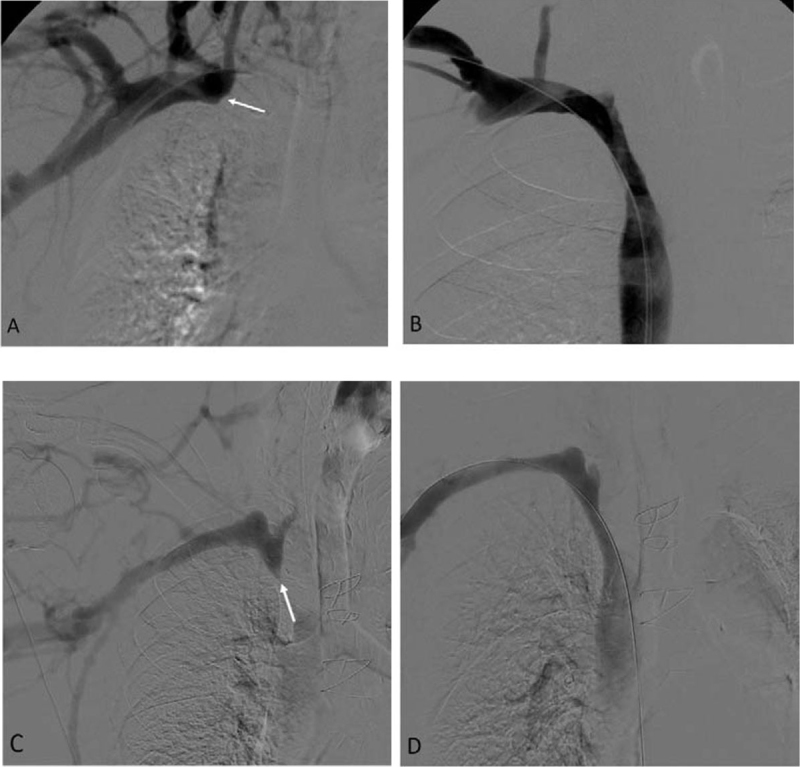
Types of occlusive lesions. **(A)** Abrupt-type occlusion (arrow) of right brachiocephalic vein in hemodialysis patient, presenting right arm and face swelling as well as thrombosis of right forearm arteriovenous graft and **(B)** Angiogram showing successful recanalization of right brachiocephalic vein occlusion. **(C)** Tapered-type occlusion (arrow) of right brachiocephalic vein in hemodialysis patient, presenting increased venous pressure during hemodialysis via right brachiocephalic arteriovenous fistula and **(D)** Angiogram showing successful recanalization of right brachiocephalic vein occlusion.

**Figure 2:**
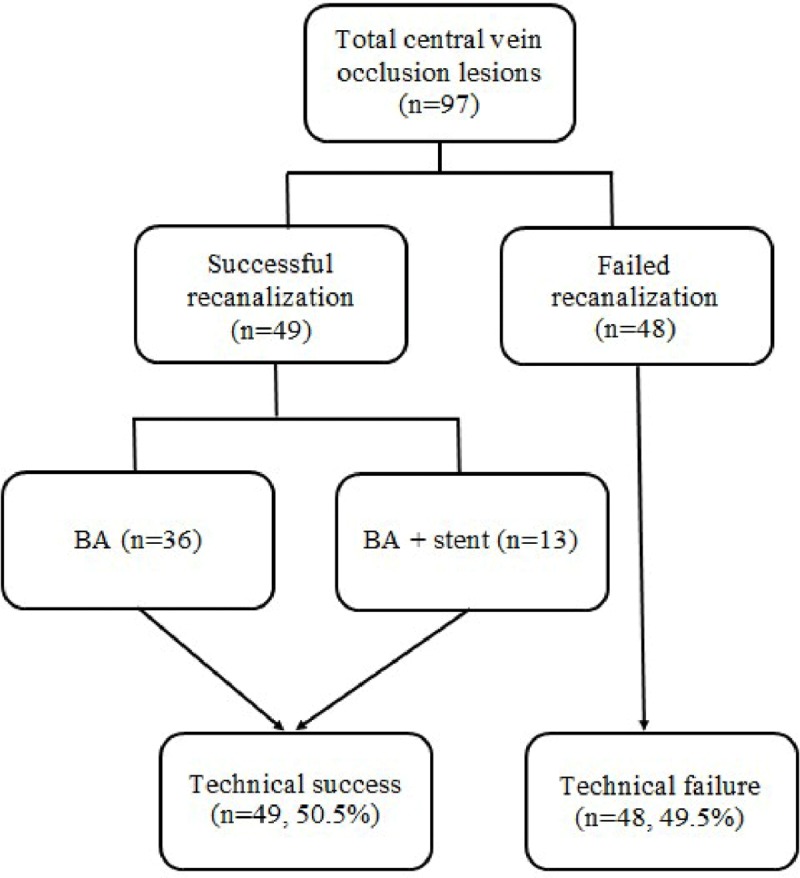
Diagram showing technical success and failure in this study. BA = balloon angioplasty.

A total of 97 hemodialysis patients with an equal number of CVO were enrolled. The patient demographic data are presented in Table 1. There were 45 males and 52 females. The mean age of patients was mean 61.2 ± 12.9 years. Hypertension is the most common underlying disease of the patients (87.6%). All patients had a previous history of ipsilateral tunnelled central vein catheter insertion with occluded central veins. Most patients in this study had hemodialysis access via arteriovenous fistula (57.7%), and most type was radiocephalic fistula (35 patients), whereas forearm graft was the most common type in patients receiving hemodialysis via arteriovenous graft (30 patients). The mean age of hemodialysis access in this study was 4.3 ± 3.1 years and mean duration between first dialysis session and presenting symptoms of dialysis dysfunction was 1.3 ± 0.4 years. The most common presenting symptom was edema of the ipsilateral upper extremity and face. The most common location of CVO was the right brachiocephalic vein (43.3%). The most common type of occlusion was the abrupt-type (71.1%), and tapered-type was found in 28.9% (Figure 1). Technical success for central vein recanalization was 50.5%, and most was achieved by antegrade recanalization (35 patients). Technical failure was caused by the inability to cross the occluded venous segment with a guidewire. In the cases of successful recanalization, most of them were treated with BA alone (36 patients) (Figure 2).

**Table 1:** Patient demographics (n = 97).

Variables	n (%)

**Age** (mean 61.2 ± 12.9 years)	
**Sex**	
Male	45 (46.4%)
Female	52 (53.6%)
**Comorbidities**	
Hypertension	85 (87.6%)
Diabetic mellitus	44 (45.4%)
Smoking	6 (6.2%)
**Previous central vein catheter insertion**	
Yes	97 (100.0%)
No	0 (0%)
**Hemodialysis access**	
Arteriovenous graft	41 (42.3%)
– Forearm graft	30 (73.2%)
– Arm graft	11 (26.8%)
Arteriovenous fistula	56 (57.7%)
– Radiocephalic type	35 (62.5%)
– Brachiocephalic type	17 (30.4%)
– Brachiobasilic type	4 (7.1%)
**Age of hemodialysis access** (mean 4.3 ± 3.1 years)	
**Duration between first dialysis session and presenting symptoms of dialysis dysfunction** (mean 1.3 ± 0.4 years)	
**Presenting symptoms**	
Arm and face swelling	75 (77.3%)
Thrombosis of hemodialysis access	10 (10.3%)
Dysfunction of hemodialysis access	8 (8.2%)
Increased venous pressure during hemodialysis	4 (4.1%)

**Figure 3:**
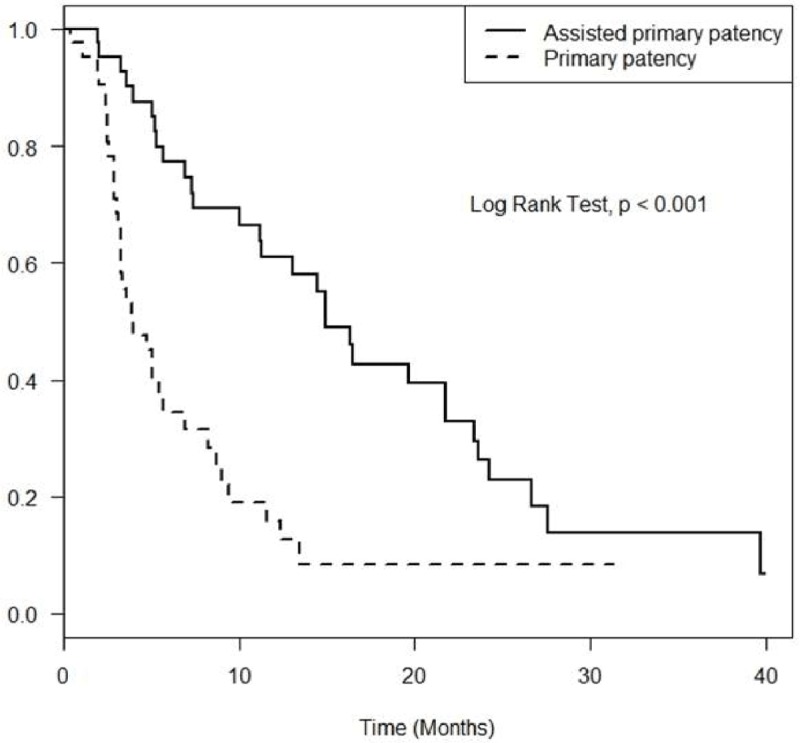
Overall primary patency and assisted primary patency rates of central vein recanalization.

**Figure 4:**
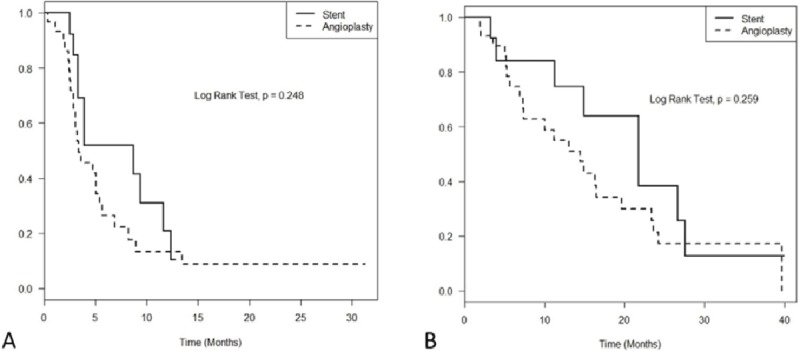
Comparing patency rates of central vein recanalization between angioplasty alone and stent groups: **(A)** Primary patency rate; **(B)** Assisted primary patency rate.

The primary patency rates of recanalized central veins at 1, 3, 6 and 12 months were 97.6%, 70.9%, 34.4% and 15.8%, respectively. The assisted primary patency rates were 100%, 95.2%, 77.3% and 61.0% at 1, 3, 6 and 12 months, respectively (Figure 3). The mean follow-up time was 14.2 months (range, 1.9–39.9 months) from initial recanalization. Comparing primary patency rates according to BA-alone and stent groups, the primary patency rates at 1, 3, 6 and 12 months were 96.6%, 64.7%, 26.6% and 13.3%, respectively, in the BA group, whereas the primary patency rates were 100%, 84.6%, 51.9% and 20.8% in the stent group, respectively. The assisted primary patency rates at 1, 3, 6 and 12 months were 100%, 93.1%, 74.5% and 54.9%, respectively, in the BA group, whereas the assisted primary patency rates were 100%, 100%, 83.9% and 74.6%, respectively, in the stent group (Figure 4). There were no statistically significant differences in primary patency (p = 0.25) and assisted primary patency (p = 0.26) between the two groups.

The details of the successful procedures are presented in Table 2. Univariate analysis was used to determine the factor-related success of recanalization of an occluded central vein. Two factors were statistically significant: age (p = 0.006) and type of occlusion (p = 0.004). The significant predictive factors are shown in Table 3. By multivariate logistic regression, age and type of occlusion were confirmed to be significant predictive factors related to success of recanalization. The success of recanalization in patients than 60 years old or older was about 2.6 times greater than in patients younger than 60 years (p = 0.034). The success of recanalization in the tapered-type occlusion was about 4.7 times greater than in the abrupt-type occlusion (p = 0.002).

**Table 2:** Univariate analysis for the recanalization success (n = 97).

Factors	Success n = 49	Failure n = 48	*p*

**Age** (year); mean (SD)	64.8 (12)	57.6 (12.8)	0.006*
**Sex**			
Male	21	24	0.616
Female	28	24	
**Comorbidities**			
Hypertension	44	41	0.522
Diabetic mellitus	23	21	0.838
Smoking	3	3	1
**Hemodialytic access**			
Arteriovenous graft	17	24	0.187
Arteriovenous fistula	32	24	
**Type of lesion**			
Abrupt-type	28	41	0.004*
Tapered-type	21	7	
**Location of lesion**			
Left BCV	22	16	0.361
Right BCV	20	22	0.541
Left SCV	1	4	
Right SCV	3	5	
Right IJV	1	0	
SVC	0	1	
Left BCV + left SCV	1	0	
Left BCV + left SCV + SVC	1	0	
**Length of lesion** (cm); mean (SD)	2.3 (0.4)	2.4 (0.6)	

* *p* < 0.05. BCV = brachiocephalic vein, SCV = subclavian vein, IJV = internal jugular vein, SVC = superior vena cava.

**Table 3:** Multivariate logistic regression for factors of success central vein recanalization.

Factors	Crude odds ratio (95% CI)	Adjusted odds ratio (95% CI)	*P*

**Age**			
<60 years	1	1	0.034*
≥60 years	2.68 (1.17,6.15)	2.61 (1.06,6.45)	
**Type**			
Abrupt-type	1	1	0.002*
Tapered-type	4.39 (1.65,11.72)	4.69 (1.63,13.47)	

* *p* < 0.05. CI = confidence interval.

## Discussion

In this study, the technical success rate of conventional recanalization was similar to that of previous studies [9,11,13,14]. The primary and assisted primary patency rates were also similar to previous reports [9,10,14]. There were no statistically significant differences of primary patency and assisted primary patency between the BA and stent groups. Patient age and types of occlusion were significant predictive factors for the success of recanalization.

We enrolled hemodialysis patients with CVO only, with a the technical success rate for recanalization of only 50.5%. By contrast, the technical success for the treatment of central vein obstruction by previous publications usually reported a combination of stenosis and CVO. Furthermore, most of the previous studies included a small number of cases with occlusion [9,13,14]. Additionally, most of the cases in each study were CVS (Table 4). This is likely the reason for the low technical success rate in our study, though the success rates of conventional recanalization of CVO cases only in previous studies ranged from 0% to 100% [9,11,13,14]. Similarly to previous studies, failure to pass the guidewire across the occluded segment was the main etiology of technical failure in our study, owing to thickened fibrous tissue and calcification [9,11,13,14]. Many advanced techniques used to improve the technical failure rates have been reported, including sharp recanalization using the back end of the guidewire or a needle [7]. Additionally, a radiofrequency guidewire was used when the conventional technique failed [15]. However, we have not used these techniques since they are less available and aggressive, leading to excess morbidity and mortality [11].

**Table 4:** Previous literature of successful recanalization of central vein occlusion.

Study (year of publication)	Number of central vein obstructions			Technical success; n (%)	

	CVO	CVS	Total	CVO	Total

**Ozyer et al. [9] (2009)**	55	101	156	46 (83.6)	147 (94.2%)
**Nael et al. [13] (2009)**	12	80	92	8 (66.7%)	82 (90%)
**Jones et al. [19] (2011)**	12	18	30	12 (100%)	30 (100%)
**Silvestre et al. [11] (2014)**	10	15	25	0 (0%)	15 (60%)
**Current study (2019)**	97	–	97	49 (50.5%)	49 (50.5%)

CVO = central vein occlusion, CVS = central vein stenosis, n = number.

The overall primary and assisted primary patency rates of endovascular recanalization are broadly variable in several studies. The results of the patency rates in this study were a bit lower than those in previous studies [4,5,6,13,14,16] since our study included only CVO cases. After recanalization, restenosis has a higher chance to occur in an occluded lesion than in a stenotic lesion [9]. Therefore, the patency time is also shorter. However, in the BA-alone group and stent group, our results were within the range of previous publications [3,4,5,6,9,10,14]. Endothelial injury can occur from BA or stenting, which results in restenosis of a central vein due to the mechanism of thrombin generation, platelet activation and upregulation of pro-inflammatory transcription factors and pro-fibrotic genes, which, in turn, cause smooth muscle proliferation, thickening of the venous intima and fibrosis [17]. Repetitive BA is necessary to maintain the patency of the central vein after successful recanalization.

Stenting can provide mechanical support for a lesion that is unresponsive to BA. It is beneficial in kinked stenosis, elastic stenosis post-PTA, sealing dissection post-PTA and maintaining patency of chronic CVO [8]. Several studies focused on bare metal stents to prolong the patency of a central vein. However, there were no significant outcomes in terms of patency rates between BA alone and primary stenting [6,10,14]. The results indicated that primary stenting does not improve long-term patency in case of CVS or CVO. Similarly to our study, Ozyer et al. [9] retorted stent placement only after failure of the primary BA. Their results showed a significantly lower primary patency rate of stenting compared with BA alone in a central vein between the two groups. Covered stent placement in central vein obstruction seemed to improve the patency rate because the graft portion provides a relatively inert and stable intravascular matrix for endothelialization as well as mechanical advantage from the frame of a bare metal stent [1,18]. Jones et al. [19] reported long-term primary patency rates of 81%, 67% and 45% at 6, 12 and 24 months, respectively, using covered stents in CVO patients. Our study did not use covered stents because of the high cost and because covered stents can cause collateral vein obstruction. Recently, few studies reported the outcome of paclitaxel-coated balloon angioplasty (PCBA) in CVS. Massmann et al. [20] showed that PCBA provided significantly better freedom from target tissue revascularization than conventional BA. Other two publications also showed similar results [21,22]. The mechanism of paclitaxel is the promotion of cell cycle arrest in the G1 phase without apoptosis of the cells that causes the reduction of the neo-intimal hyperplasia of the venous wall [22]. However, there are still no data showing the efficacy of primary PCBA in chronic CVO.

A review of previous literature revealed that the predictive factors for successful recanalization in CVO only were not reported because most publications included both CVS and CVS. On the basis of our experience and the literature review, this current study is the first to report the predictive factors for successful recanalization in CVO. Patient age and the type of occlusion were significant predictive factors related to the success of recanalization.

Patients aged ≥60 years have a higher rate of recanalization, but the explanation of this result is not clear. A possible reason may be that older patients could not tolerate the symptoms and came for early treatment. The tapered-type occlusion had a high technical success rate for recanalization. We believe that the tapered-type may indicate a recent occlusion that maintained microchannels that were not seen on the angiogram. Thus, the guidewire could cross the occluded segment with less resistance.

There are two strengths of this study. First, this study enrolled only CVO cases. which caused homogeneity of the results. Second, we evaluated the predictors for success in CVO recanalization, which were not determined in previous studies. However, this study has a limitation that was a retrospective study at a single center. A prospective design with a multicenter approach should be performed to confirm our results.

In summary, endovascular treatment of CVO in hemodialysis patients using the conventional technique, there was no significant difference in primary patency and assisted primary patency between BA alone and stenting. Patients aged ≥60 years and tapered-type occlusion were two predictors for successful recanalization in CVO.
